# The Dual Role of Natural Organic Matter in the Degradation of Organic Pollutants by Persulfate-Based Advanced Oxidation Processes: A Mini-Review

**DOI:** 10.3390/toxics12110770

**Published:** 2024-10-23

**Authors:** Dan Luo, Hansen Lin, Xingzhen Li, Yu Wang, Long Ye, Yuebang Mai, Peihao Wu, Zhuobiao Ni, Qingqi Lin, Rongliang Qiu

**Affiliations:** 1 Guangdong Laboratory for Lingnan Modern Agriculture, Guangdong Provincial Key Laboratory of Agricultural & Rural Pollution Abatement and Environmental Safety, College of Natural Resources and Environment, South China Agricultural University, Guangzhou 510642, China; luod@stu.scau.edu.cn (D.L.); linhansen@stu.scau.edu.cn (H.L.); lxz@stu.scau.edu.cn (X.L.); nizhuobiao@scau.edu.cn (Z.N.); qiurl@scau.edu.cn (R.Q.); 2 Engineering and Technology Research Center for Agricultural Land Pollution Integrated Prevention and Control of Guangdong Higher Education Institute, College of Resources and Environment, Zhongkai University of Agriculture and Engineering, Guangzhou 510225, China; 3 Guangdong Provincial Academy of Building Research Group Co., Ltd., Guangzhou 510510, China; longye031@163.com (L.Y.); maiyuebang@163.com (Y.M.); wupeihao@gdjky.com (P.W.); 4 School of Environmental Science and Engineering, Sun Yat-Sen University, Guangzhou 510006, China

**Keywords:** persulfate, advanced oxidation process, degradation, organic pollutants, natural organic matter

## Abstract

Persulfate-based advanced oxidation processes (PS-AOPs) are widely used to degrade significant amounts of organic pollutants (OPs) in water and soil matrices. The effectiveness of these processes is influenced by the presence of natural organic matter (NOM), which is ubiquitous in the environment. However, the mechanisms by which NOM affects the degradation of OPs in PS-AOPs remain poorly documented. This review systematically summarizes the dual effects of NOM in PS-AOPs, including inhibitory and promotional effects. It encompasses the entire process, detailing the interaction between PS and its activators, the fate of reactive oxygen species (ROS), and the transformation of OPs within PS-AOPs. Specifically, the inhibiting mechanisms include the prevention of PS activation, suppression of ROS fate, and conversion of intermediates to their parent compounds. In contrast, the promoting effects involve the enhancement of catalytic effectiveness, contributions to ROS generation, and improved interactions between NOM and OPs. Finally, further studies are required to elucidate the reaction mechanisms of NOM in PS-AOPs and explore the practical applications of PS-AOPs using actual NOM rather than model compounds.

## 1. Introduction

Large amounts of organic pollutants (OPs), including traditional and emerging compounds, are intentionally or accidentally discharged into the environment, posing extensive risks to the ecosystem and human health. Therefore, there is a pressing need for efficacious methods to control and degrade these pollutants [1,2]. Among various technologies, advanced oxidation processes (AOPs) are widely employed in the treatment of contaminated water and soil matrices due to their advantages, such as rapid reaction, wide applicability, high efficiency, and being environmentally friendly [3,4,5]. AOPs are in situ chemical oxidation technologies that generate reactive oxygen species (ROS) for pollutant degradation by the addition of various oxidants [6,7]. ROS can be generated through various methods, depending on the selection of activators and oxidants [8,9]. Commonly utilized oxidants in AOPs include hydrogen peroxide, permanganate, ozone, and persulfate (PS). However, the AOPs that employ hydrogen peroxide require stringent pH conditions. The oxidation process using permanganate has limited effectiveness in degrading chlorinated alkanes and certain aromatic compounds, while the application of ozone for soil remediation poses significant challenges. In comparison, PS emerges as a competitive alternative due to its broader applicability and ease of operation [10,11]. PS can be activated by heat, alkali, electricity, transition metals, metal composite materials, and modified carbon-based catalysts, producing free radicals including sulfate radicals (SO_4_^●−^), hydroxyl radicals (^●^OH), and superoxide anion radicals (O_2_^●−^) [10,12,13,14,15]. Peroxymonosulfate (PMS) and peroxydisulfate (PDS) release a significant amount of SO_4_^●−^ and ^●^OH radicals through either homolytic or heterolytic cleavage of the peroxide bond. However, their activation processes and reactivity towards organic functional groups differ due to distinctions in the symmetry of the peroxide bond [16]. In addition to free radical reactions, PSs can also remove OPs through other non-radical reactive oxidants generated from high valency metals and single electron transfer [17,18,19,20]. Free-radical-based AOPs exhibit higher removal efficiencies for OPs within a relatively short contact time, while non-free-radical AOPs experience less interference from water matrices, making them suitable for treating complex wastewater [11,21]. 

In previous studies, natural organic matter (NOM) was often present as a pollutant or was considered to inhibit the oxidation of OPs [22,23,24,25]. However, in practice, the influence of ubiquitous NOM in the environment may be more complicated. For instance, high concentrations of humic acid (HA) inhibit the elimination of OPs by permanganate oxidation [26], whereas NOM at low concentrations slightly promotes the degradation of OPs via the formation of a reactive Mn species from permanganate by the phenolic group in the NOM [27]. Moreover, in a study on the degradation of nitroimidazoles (NZs) in a UV/peroxymonosulfate AOP, the rate of degradation increased under different dissolved organic matter (DOM) conditions, indicating a positive correlation between enhancement and the total electron capacity of the NOM [28]. Recent studies have shown that NOM can have dual effects in degrading micro-pollutants in PS-AOPs, depending on the concentrations and structures of the NOM [29,30,31,32]. The complex structures of the NOM can both promote and inhibit ROS generation in PS-AOPs; an optimal concentration range can promote pollutant transformation (Table 1), but exceeding this range can have adverse effects [33,34]. Overall, NOM induces both promotion and inhibition simultaneously in PS-AOPs, with the effectiveness of the process depending on finding the right balance between these dual effects [35].

NOM is commonly found in water, soil, and sediment. It originates from various sources such as humus, fresh animal and plant residues, root exudates, and microbial metabolites. NOM plays a crucial role in the migration, transformation, and biotoxicity of Ops [36,37]. Additionally, biochar—heat-treated plant biomass—is a prevalent source of pyrogenic dissolved organic carbon in the environment [38]. The composition of NOM is greatly influenced by its sources and the biogeochemical processes it undergoes [39]. NOM has a complex structure, consisting of condensed components such as HA, fulvic acid (FA), humin substances, and lignin, as well as amorphous components such as fatty acids, peptides, and phenolic compounds [40,41]. Furthermore, NOM contains numerous hydrophilic or hydrophobic functional groups, including alkyl chains, aromatic rings, hydroxyl groups, and carbonyl groups, which impact its interactions with other substances [24,42]. In particular, phenols and quinones are key redox-active components that play a significant role in electron transfer processes, affecting the behavior of oxidants and pollutants [43,44].

**Figure 1:**
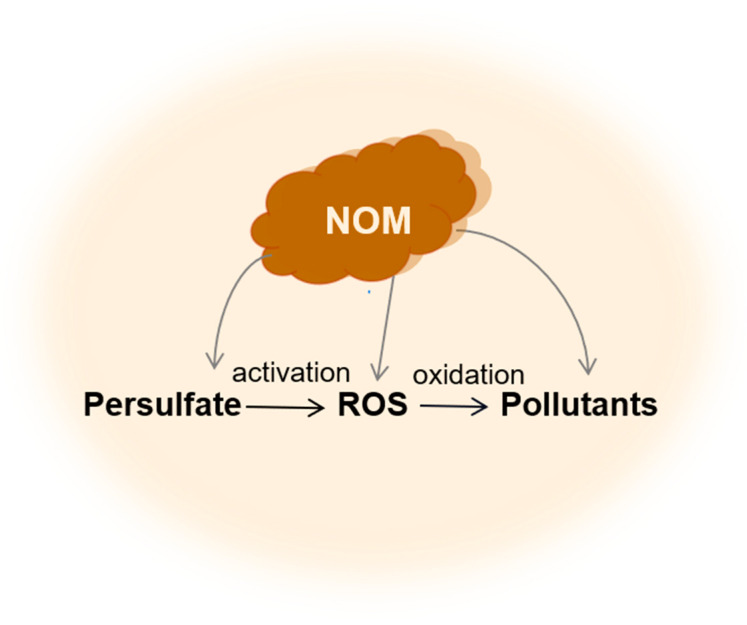
Reaction pathways of NOM involved in the degradation of OPs by PS-AOPs.

NOM is ubiquitous in the environment, and the efficacy of PS-AOPs in removing OPs is significantly influenced by the presence of NOM. However, the processes underlying the various roles of the NOM in the degradation of OPs in aquatic and soil matrices via PS-AOPs remain poorly understood. Therefore, it is necessary to gain a deeper understanding of the impact of NOM on PS-AOPs. This review summarizes the inhibitory (Section 2) and promoting (Section 3) effects of NOM on the interaction between PS and its activators, the fate of ROS, and the transformation of OPs in PS-AOPs occurring in water and soil matrices (Figure 1). Additionally, the challenges and prospects related to the dual effects of NOM in PS-AOPs are discussed (Section 4).

**Table 1:** Effects of NOM on the degradation of OPs in PS-AOPs.

Reference	Degradation Efficiency of Pollutants	NOM Effects	PS-AOP Type	NOM Type	Target Pollutant
[45]	The removal efficiency of oxytetracycline was reduced by approximately 20%, and the reaction rate constant kobs decreased from 0.182 to 0.038 min^−1^ with the addition of 10 mg·L^−1^ HA.	Inhibiting effect	3DP-HPC@CoAl-LDH ^1^/PMS	Humic acid (HA)	Oxytetracycline
[46]	As the NOM concentration increased from 1 to 5 mg·L^−1^, the removal rate of 4-chloro-3,5-dimethylphenol decreased from 3.85 × 10^−4^ to 1.38 × 10^−4^ s^−1^.		UV/PDS	Humic acid (HA)	4-Chloro-3,5-dimethylphenol
[47]	The degradation rate of ofloxacin in the GA/Fe(III)/PS system increased by more than 80% compared to the control without GA addition.	Promoting effect	GA/Fe(III)/PDS	Gallic acid (GA)	Ofloxacin
[34]	The removal rate of naphthalene reached 71.78% in the Ilex extra/Fe(II)/PDS system, which was 1.86-fold higher than for the Fe(II)/PDS system (38.58%).		Ilex extra/Fe(II)/PDS	Ilex extra	Naphthalene
[48]	The degradation of bisphenol S by PMS was significantly enhanced by EGCG at pH 3.0–7.0, but inhibited at pH 8.0–10.0.	Dual effects	EGCG/PMS	Epigallocatechin-3-gallate (EGCG)	Bisphenol S
[49]	The removal rate of ibuprofen increased from 71.9% to 77.3% as HA concentration increased from 0 to 5 mg·g^−1^. However, a decrease (74.8% to 56.8%) was observed when HA concentration increased from 10 to 50 mg·g^−1^.		Fe(II)-SP ^2^/PS	Humic acid (HA)	Ibuprofen

Notes: ^1^ 3DP-HPC@CoAl-LDH: CoAl-layered double hydroxide nanoparticles (CoAl-LDH) were immobilized on the framework of 3D printed hierarchical porous ceramics (3DP-HPC) to form a composite material that could be used to activate PMS. ^2^ Fe(II)-SP: The chelation between pyrophosphate (SP) and Fe(II) can significantly activate PS and promote the degradation of IBP in soil systems.

## 2. The Inhibitory Effects and Mechanisms of NOM on OP Removal by PS-AOPs

### 2.1. NOM Prevents the Activation of PS

The activation of PS results in the generation of ROS such as SO_4_^●−^ and ^●^OH, which are essential for breaking down OPs. However, during these reactions, the NOM may obstruct the interaction between PS and its activators, thus slowing the activation process and hindering the reduction of OPs.

#### 2.1.1. Electrostatic Repulsion and Spatial Impedance

**Figure 2:**
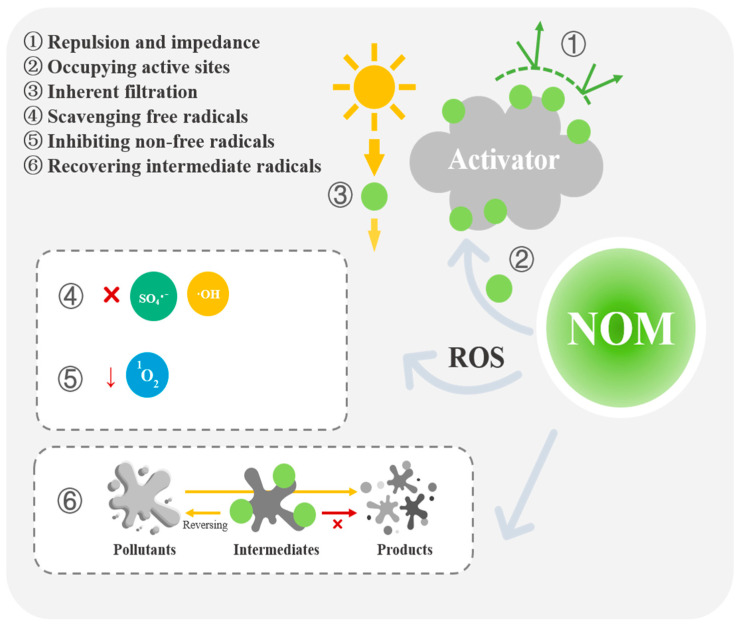
Inhibitory effects of NOM on OP removal in PS-AOPs.

NOM molecules have complex structures and contain numerous atoms or groups that can create steric hindrance, i.e., hindering the effective contact between the activator and PS (Figure 2 ①). The addition of HA (a NOM model) to a PDS/carbon nanotube (CNT) system significantly inhibited the degradation of sulfamethoxazole (SMX), with this inhibitory effect becoming more pronounced as the concentration of HA increased. This was attributed to electrostatic repulsion and steric hindrance effects [50]. The CNTs activated the PDS through non-radical means, with the PDS initially forming surface complexes with the CNT active sites. The PDS then reacted with target pollutants or underwent electron transfer within the complex, leading to its decomposition into SO_4_^2−^. By enhancing the spatial and electrostatic repulsion among PDS molecules, the adsorbed HA indirectly restricted the PDS from approaching the activator surface, hindering the interaction between PDS and the CNTs and preventing the formation of surface complexes [51,52].

#### 2.1.2. The Active Sites of Solid Catalysts Occupied by NOM

The presence of NOM on the surface of the catalyst used for PS activation means that it can adsorb and occupy active sites, leading to a decrease in catalytic efficiency and a subsequent reduction in the production of SO_4_^●−^ and other active substances (Figure 2 ②) [53]. Incorporating transition metals into metal oxides is a convenient approach to introduce oxygen vacancies (OVs), which can enhance PDS adsorption on the catalyst surface and form surface-activated complexes through weak interactions. Complexes with OVs exhibit strong oxidation abilities and can acquire electrons from electron-rich pollutants such as bisphenol A (BPA). Consequently, the O-O bonds in the PDS are cleaved, separating it from the complex and converting it into SO_4_^2−^. However, when utilizing hollow OV-rich ZnCo_2_O_4_ nanomaterials for PDS activation, high concentrations of HA compete with the PDS for active sites on the nanomaterial surface, impeding the formation of surface-activated complexes and slowing the degradation reaction rate of the pollutant BPA [54]. It has also been reported that aromatic groups in HA may competitively occupy PDS activation sites due to π-π bonding, hydrophobicity, and hydrogen bonding interactions with CNTs [52].

#### 2.1.3. The Inherent Filtration Impact of NOM

NOM in water bodies can act as an “internal UV filter”, competing with oxidants like PS and absorbing photons (Figure 2 ③). The chromophores in NOM have strong absorption capabilities in the UV and near-UV spectrum, which can impede light transmission, reduce UV transmittance, and decrease photon absorption efficiency. This decreases the utilization of light for the generation of ROS [55,56,57]. A study demonstrated that NOM significantly reduces the degradation efficiency of azathioprine (AZA) when comparing direct UV photolysis, UV/H_2_O_2_, and UV/PS methods. This is mainly due to the ability of NOM to absorb UV photons, thereby decreasing the solution’s permeability to UV irradiation and weakening its photocatalytic effects, ultimately leading to lower ROS production [58]. Similarly, in an Fe(II)/citrate/UV/PMS system, different concentrations of NOM (0.1, 0.5, 1, and 5 mg·L^−1^) lead to varying absorbances by Fe(III)-citrate complexes (5%, 35%, 65%, and 375%, respectively). The high absorbance capacity of the NOM significantly decreases the absorption of light by the Fe(III)-citrate complex, resulting in a decline in the Fe(II) regeneration rate and inhibiting the degradation rate of carbamazepine (CBZ) [59].

### 2.2. Suppression of ROS by NOM

The presence of NOM not only hinders the direct interaction between activators and PS but also competes with target pollutants for the ROS, thereby impacting the degradation efficiency of PS-AOPs [12,60].

#### 2.2.1. NOM Effectively Scavenges Free Radicals and ROS

In NOM structures, certain active groups show an increased reactivity towards free radicals; that is, NOM acts as a scavenger for reactive free radicals and a sink for SO_4_^●−^ and ^●^OH (Figure 2 ④) [61]. Xie et al. utilized UV/PS to eliminate 2-methylisoborneol (2-MIB) and geosmin, finding that NOM competes with these pollutants in reacting with free radicals, leading to a decrease in the concentration of free radicals and a subsequent reduction in the removal efficiency. The NOM primarily affects the clearance of ^●^OH, with a reaction rate constant between NOM and ^●^OH of 1.6–3.3 × 10^8^ Mc^−1^·s^−1^, which is over ten times higher than that between NOM and SO_4_^●−^. Therefore, higher NOM doses slow the consumption of SO_4_^●−^ by the NOM, which is the main ROS involved in pollutant degradation [62,63]. The concentration of SO_4_^●−^ also impacts the steady-state concentration of ^●^OH because SO_4_^●−^ can generate ^●^OH by reacting with H_2_O and OH^−^ (Equations (1) and (2)) [64,65]. Different types of NOM exhibit varying effects on free radical removal, with some inhibiting SO_4_^●−^ from scavenging on stable oxidation intermediates or readily reducible compounds (Equation (3)) [66]. NOM with more aromatic groups and antioxidant moieties show an increased reactivity towards SO_4_^●−^ [67]. Soil-derived humic substances, due to their large molecular size and high phenolic content, scavenge more SO_4_^●−^ than other humic substances [68].

SO_4_^●−^ + H_2_O → ^●^OH + H^+^ + SO_4_^2−^ (1)

SO_4_^●−^ + OH^−^ → ^●^OH + SO_4_^2−^ (2)

DOM + SO_4_^●−^ → SO_4_^2−^ + RIs_DOM_ (3)

#### 2.2.2. Inhibition of Non-Free-Radical Active Substance Generation by NOM

In addition to generating ROS as free radicals, PS-AOPs can also produce singlet oxygen (^1^O_2_) and highly active substances such as high valency metals and their oxides (e.g., Co (IV) and high valency nickel oxides) [19,69,70]. The presence of NOM in the system can inhibit the formation of non-free-radical active substances, thereby reducing their efficiency in degrading pollutants (Figure 2 ⑤). To enhance the degradation efficiency of acetaminophen (APAP), a catalyst consisting of single cobalt atoms dispersed on 2D carbon nanoplates (SA-Co CNP) was synthesized, which was then reacted with PMS. Cobalt atoms are considered crucial active sites for PMS activation, leading to the generation of abundant ROS—primarily ^1^O_2_—which are involved in APAP degradation. However, HA competes with ^1^O_2_ during degradation and significantly hampers APAP degradation. The addition of 1 mM HA resulted in a decrease in the kinetic constant for APAP degradation from 0.51 to 0.14 min^−1^ in the presence of 0.1% SA-Co CNP/PMS [71].

### 2.3. Conversion of Pollutant Intermediates to Their Parent Compounds by NOM

NOM not only reduces the competitiveness of the ROS but can also convert the intermediate products of pollutants back to their original compounds (Figure 2 ⑥) [72]. During the photochemical degradation process of sulfonamide and benzene amine compounds, the antioxidant groups present in the DOM (such as electron-donating phenolic aldehyde groups) can impede compound oxidation by reducing the free radical intermediates back to their parent compounds [73,74]. Research by Cheng et al. showed that incorporating 1.0 mg·L^−1^ of DOM results in an 88% decrease in the degradation of the micro-pollutant adenine (ADN) compared to the control, with approximately half of this reduction attributed to the regeneration of ADN radicals (ADN(-H)^●^) into the parent compound. The inhibitory effect and rate constant for quenching ADN (-H)^●^ are closely related to the antioxidant properties exhibited by the DOM, such as its phenolic group content. The rate constant for the quenching of ADN (-H)^●^ by DOM ranges from 0.39 × 10^7^ to 1.18 × 10^7^ Mc^−1^·s^−1^ based on its antioxidant characteristics [33,75]. Usually, NOM with excessive phenoxy and phenolic groups can easily reduce the intermediates to their parent compounds. This is potentially attributed to the inherent characteristics of NOM [29]; however, further studies on the transformation mechanisms are needed.

## 3. Promoting Effects and Mechanisms of NOM on OP Removal by PS-AOPs

Although NOM, such as HA, in the environment can inhibit the effectiveness of PS-AOPs, other types of NOM, such as natural polyphenols, are often introduced in studies to enhance the activation process. Natural polyphenols contain hydroxyl, phenol, and quinone groups, which are crucial for electron transfer within the system [76,77,78]. Polyphenols like GA and catechin (CAT) facilitate the reduction of transition metals by forming complexes with iron and generating reductive quinone intermediates [79,80]. Tannic acid (TA), another common natural polyphenol with hydroxyl groups and benzene structures, can form coordination bonds with metal ions, allowing TA–modified catalysts to undergo strong interfacial interactions and enhancing the performance of the oxidation system [81,82]. Table 2 provides an overview of studies that explore the role of NOM in promoting PS-AOPs.

### 3.1. NOM Enhances the Catalytic Effectiveness of Activators

Nano-Fe^0^ has the propensity to agglomerate and oxidize, but NOM can serve as a stabilizer that enhances its dispersion and stability. For example, NOM can assist in the production of graphene nanoshell–encapsulated nano-Fe^0^, resulting in a core–shell structure. Additionally, HA-coated nanoparticles can improve their stability against aggregation by modifying the surface charge state of particles, similar to the role of surfactant molecules in the “green” surface coatings of nanoparticles [87,88]. Furthermore, research has shown that TPs can act as reducing agents to convert Fe(II) and facilitate the preparation of nano-Fe^0^ [84].

**Figure 3:**
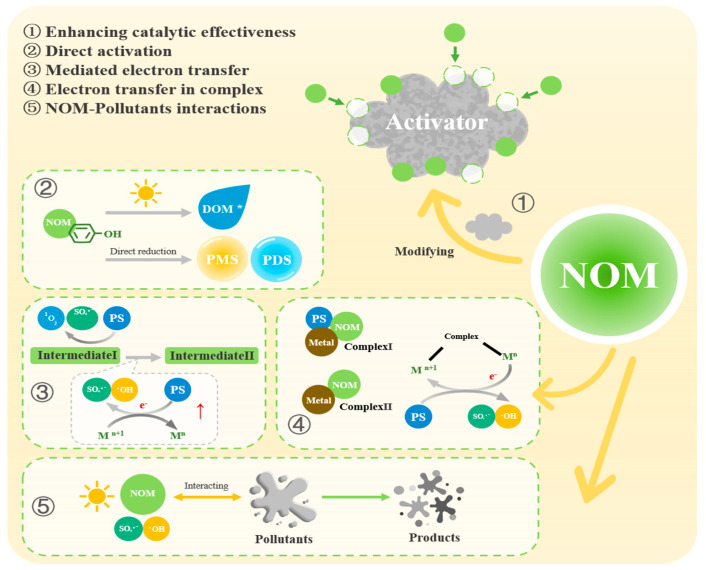
Promotion effects of NOM on OP removal in persulfate-based advanced oxidation processes.

Studies have shown that the conversion of agricultural and forestry waste into biochar can enhance catalyst performance and aid in the degradation of pollutants (Figure 3 ①) [89,90,91]. Researchers have utilized FA, HA, and hydrochar-derived dissolved organic matter (hyDOM, biochar derived from the hydrothermal treatment of natural biomass which releases large amounts of DOM) to create Co-Fe bimetallic catalysts (CoFeO). The impact of these organic materials on the performance of CoFeO catalysts and the oxidative degradation of BPA was assessed. Results showed that the effect of these organic materials on the CoFeO/PMS system varied depending on the DOM concentration. Introducing low amounts of organic matter improved the physical and chemical properties of CoFeO, leading to enhanced BPA degradation in the CoFeO/PMS system. The physical and chemical properties of CoFeO were further characterized and changes in morphology and pore structure were observed. Specifically, the length and diameter of the composite material decreased while the average pore width and volume significantly increased, providing more active catalytic sites. Additionally, the increase in CoFeO functional groups and surface OVs enhanced the electronic conductivity, while in the hyDOM-CoFeO and FA-CoFeO composites, the incorporation of HA compounds accelerated the reduction of Co(III)/Fe(III) to Co(II)/Fe(II), resulting in a significant enhancement of activation performance [92].

### 3.2. NOM Contributes to the Generation of ROS

#### 3.2.1. NOM Directly Generates ROS

*Photolysis of DOM.* Photoactivated PS can generate ROS, while NOM can undergo direct or indirect photolysis to eliminate target pollutants by converting light energy into an excited state (^3^DOM*) and generating ROS (^●^OH, ^1^O_2_, Figure 3 ②) [93]. Studies have demonstrated that the indirect photodegradation of ^3^DOM* can outperform direct photodegradation in photochemical reactions [94]. The relative contributions of free radicals and DOM* to direct and indirect photolysis vary for different pollutants. For example, ^1^O_2_ plays a crucial role in the indirect photodegradation of sulfathiazole, while triplet chromophoric DOM (^3^CDOM*) is essential for the indirect photodegradation of sulfamerazine. The composition and source of chromophoric DOM (CDOM) can impact its photolysis efficiency; DOM with a higher molecular weight and more aromatic groups exhibits enhanced photochemical properties, resulting in higher levels of ^●^OH, ^1^O_2_, and ^3^CDOM*, thereby enhancing the indirect photodegradation effect [95].

*Direct reduction of PS by phenols.* The functional groups of NOM, such as peroxides, phenols, acids, and enzymes, exhibit redox activity and play a crucial role in degrading pollutants through various reaction mechanisms (Figure 3 ②) [85]. Additionally, PDS can be directly activated by electron-providing phenolic components present in the NOM [96]. Ahmad et al. conducted a study using pentachlorophenol to distinguish between phenol- and base-activated PS. Their findings demonstrated that when phenolic substances were in their dissociated anionic form (phenoxide), the PDS could be activated through one-electron reduction to produce SO_4_^●−^, similar to Fenton-like reactions [97]. In another study on the reaction between PS and humic substances (HS), Kim et al. observed a higher PS consumption in the presence of standard HS and HS model compounds compared to a control system with only benzene. They also noted a linear correlation between PS consumption and phenol content in humus [68].

#### 3.2.2. NOM Generates Intermediates and Complexes for the Production of ROS

*Quinone intermediates.* The role of NOM in promoting reactions is complex, involving various quinones or quinone-like compounds that act as crucial intermediates [47,68]. These intermediates can serve as either electron acceptors or donors, aiding in electron transfer, self-circulation, and promoting iron circulation (Figure 3 ③). Consequently, this process results in the continuous production of oxidants like SO_4_^●−^ [98]. For clarity, benzoquinone (BQ) is commonly used as a representative quinone.

In the presence of transition metal ions that can be recycled between two oxidation states, a semiquinone radical-dependent Fenton-like mechanism may occur. BQ contains two carbonyl groups (C=O) attached at the para-positions of the benzene ring. BQ can undergo self-condensation or decomposition by converting the carbonyl groups to hydroxyl groups (-OH), resulting in the reduced hydroquinone (HQ) structure. The HQ, which is unstable within the system, can be reoxidized back to BQ, releasing electrons in the process. In iron-activated PS-AOPs, the oxidation of HQ may be catalyzed by sulfate radicals or other oxidizing substances such as Fe^3+^. The electrons released during this oxidation can promote the reduction of iron to regenerate Fe^2+^, enabling an efficient catalytic cycle (Equations (4)–(6)) [98,99]. The BQ and HQ can generate a semiquinone radical (SQ^●−^) through comproportionation, which activates the PS (Equations (7) and (8)). SQ^●−^ radicals are highly reactive species containing a hydroxyl group, a carbonyl group, and an unpaired electron. These radicals can either donate electrons to regenerate BQ (Equations (9)–(11)) [85] or directly interact with pollutants and other free radicals to aid in pollutant degradation [100].

HQ + M^n+1^ → SQ^●−^ + M^n^ (4)

SQ^●−^ + M^n^ → BQ + M^n+1^ (5)

BQ + M^n^ → SQ^●−^ + M^n+1^ (6)

HQ + BQ → 2SQ^●−^ (7)

HQ + SO_4_^●−^ → SQ^●−^ (8)

SQ^●−^ + SO_4_^●−^ → BQ + SO_4_^2−^ (9)

2SQ^−^ → HQ + BQ (10)

SQ^●−^ + O_2_ → BQ + O_2_^●−^ (11)

The rate and type of quinone intermediates formed in heterogeneous catalytic systems can be influenced by the substituent groups and their positions in the NOM. Ortho- and meta-substituted phenols are more effective than para-substituted phenols, particularly in the case of cresols and methoxyphenols [101].

Upon reaction with PMS, BQ generates ^1^O_2_ by decomposing PMS. BQ can be considered a ketone containing two carbonyl groups. Initially, the PMS (HSO_5_^−^) attacks the carboxyl carbon atoms of BQ, forming a peroxide adduct intermediate, which further converts into a dioxirane intermediate. This dioxirane intermediate is then acted upon by two ionized PMS ions (SO_5_^2−^), resulting in the production of ^1^O_2_ and the regeneration of BQ. This process facilitates the oxidation of pollutants via a non-radical pathway [102].

*Other kinds of intermediates.* In addition to the effects of phenols, quinones, and semiquinones, NOM can also generate other organic by-products that facilitate oxidation–reduction processes (Figure 3 ③). For instance, the phenolic groups in DOM can be readily oxidized by SO_4_^●−^ to form phenoxy radicals [103]. Some phenoxy groups containing electron-withdrawing groups, like carboxyl groups, can serve as secondary oxidants (also known as secondary reaction intermediates), leading to the development of novel pathways for the degradation and transformation of pollutants [66].

The oxidation intermediates of NOM can undergo electron transfer from Fe(III) to HSO_5_^−^, thereby facilitating the degradation of pollutants [34]. For example, the addition of a small amount of GA—a representative of natural polyphenols—into the Fe(III)/PMS system can have lasting impacts on the Fe(II) cycle. Aromatic radicals and ring-opening products, such as the polyhydroxycyclohexadienyl radical (poly-HCD^●^) and poly-hydroxybenzoic acid (poly-HBA)—generated during the oxidation of GA—can efficiently reduce Fe(III), continuously activate PMS, produce SO_4_^●−^ and ^●^OH, and accelerate the degradation of 2,2′, 4,4′-tetrabromodiphenylether. Replacing GA with a green tea extract produced a similar degradation effect, suggesting that the oxidation intermediates of natural polyphenols are essential in the iron redox cycle [104].

The phenolic and quinone compounds found in soil organic matter (SOM) trigger the formation of SQ^●^. As the SOM decomposes, it produces alkyl fragments (RH), which then interact with either ^●^OH or SO_4_^●−^ to form R^●^ through H-abstraction processes. R^●^ can activate PS to generate SO_4_^●−^ (with a second-order reaction rate constant of approximately 1.5 × 10^5^ M^−1^·s^−1^) via electron transfer. The formation of R^●^ can also lead to the production of RO_2_^●^ by reacting with oxygen (O_2_), thereby initiating a free radical chain reaction. Subsequently, RH is further oxidized by H-abstraction to regenerate R^●^. When the rate at which R^●^ activates PS to produce SO_4_^●−^ exceeds the rate at which the SOM consumes SO_4_^●−^, then the SOM can enhance pollutant degradation. In the presence of PS in soil, SOM plays a crucial role in the decomposition of PS. Free radicals are generated during this process. In soils with different levels of SOM content (low, medium, and high), the primary reactive species are ^●^OH, SO_4_^●−^/^●^OH/R^●^, and R^●^ radicals, respectively. The transformation of these species is influenced by the SOM level [100].

*Complexation products.* In transition-metal-activated PS-AOPs, the reduction of high valency metals to low valency metals is a crucial step in ROS generation [53,105]. NOM enhances this reduction process in two distinct ways (Figure 3 ④). Firstly, in the presence of both BQ and PMS, an active complex (BQ-PMS*) is formed on the surface of Fe_3_S_4_, initiating an internal electronic cycling process “PMS—BQ—S—Fe—PMS”. If BPA is present, it is targeted by BQ-PMS*, which extracts electrons from BPA and transfers them to the surface of Fe_3_S_4_, causing valence changes in S and Fe. These electrons are then transferred to BQ through PMS*, enabling BQ to provide electrons to Fe_3_S_4_ to reduce the S species. The low valency S species then further reduce Fe(III) to Fe(II), with Fe(II) transferring electrons to the PMS part of the complex, resulting in the continuous activation of BQ-PMS* [106]. Secondly, NOM can chelate with Fe or other metals to form Fe(III)-NOM complexes, which reduces the redox potential of Fe(III)/Fe(II) [107,108]. These complexes facilitate the reduction of Fe(III) to Fe(II) through internal complex electron transfer, as well as direct reduction by electron transfer. In the Fe(III)/GA/PMS system, GA complexed with Fe(III) focuses electron density on the coordination site, leading to a redistribution of the electronic structure of GA. Fe(III) primarily coordinates with GA at the phenolic hydroxyl sites, with the C atoms in the ring and the O atoms in the hydroxyl and carboxylic groups susceptible to electrophilic attack for electron release. Simultaneously, GA undergoes hydroxyl self-oxidation, releasing electrons that continuously reduce Fe(III) in the complex, thereby regenerating Fe(II) to activate PMS [80]. Additionally, the addition of NOM promotes the reduction and dissolution of solid-phase Fe-minerals. Extracted TP can directly complex with the surface Fe(III) of insoluble solid Fe species, increasing the Fe(II) content, weakening bonds between the reduced Fe and adjacent Fe, accelerating Fe release into the aqueous solution, and inducing PDS activation [109].

### 3.3. The Interaction Between NOM and Pollutants

Higher fractions of NOM can promote the adsorption and immobilization of OPs on clay surfaces, affecting their reaction with oxidants (Figure 3 ⑤) [43]. Various mechanisms such as adsorption, hydrophobic distribution, and van der Waals associations can facilitate interactions between the NOM and pollutants. Oxygen-doped porous graphite carbon nitride (OCN), prepared via thermal polymerization, can activate PS to degrade persistent pharmaceuticals like CBZ. Incorporating HA into the OCN/PMS system considerably enhances CBZ degradation, with the degree of enhancement correlating with the HA concentration. The degradation kinetics of CBZ in photocatalytic reactions follow a pseudo-first-order equation. As the HA concentration increases from 5 to 30 mM, the rate constant (Ka) for CBZ degradation increases from 0.0235 to 0.0697 min^−1^. After 1 h of CBZ and HA mixing, the three-dimensional fluorescence peak of HA shifts from the initial HA-like region to a lower excitation and emission wavelength region, possibly due to interactions between the hydrophobic group of CBZ and the aliphatic/aromatic components in HA [86]. Additionally, interactions of CBZ with DOM may involve hydrogen bonding and/or π-π bonding [110]. After a 3 h photocatalytic reaction, CBZ is nearly completely degraded, and fluorescence peaks reappear in the HA-like region. This can be explained by the interaction of HA with pollutants and their co-adsorption or accumulation on catalysts to enhance CBZ degradation [86].

## 4. Conclusions and Prospects

Due to its high redox potential, chemical stability, long half-life, and environmental-friendliness, PS is extensively used in environmental applications for organic pollutant remediation. The ROS generated in PS-AOPs, such as SO_4_^●−^, ^●^OH, O_2_^●−^, ^1^O_2_, and NOM^●^, play a crucial role in degrading OPs by facilitating their transformation. In practical applications, PS-AOPs are significantly influenced by the ubiquitous presence of NOM in the environment, which complicates the overall situation. NOM exerts dual effects on PS-AOPs, both inhibiting and promoting the process. Specifically, NOM can hinder the interaction between PS and its activators [51,52,54,58,59], suppress ROS generation [62,63,71], and alter pollutant transformation pathways [33,75]. Conversely, NOM can also directly reduce PS while enhancing activator efficiency by promoting ROS production and regenerating activators [80,97,98,99,100,106]. Furthermore, the interactions between NOM and pollutants facilitate pollutant degradation [86]. Importantly, NOM can undergo redox reactions during PS-AOPs. Concurrently, electron transfer may occur, allowing the NOM to participate in and influence PS-AOPs. Notably, the functional groups present in the NOM can indicate its electron exchange capacity [111], particularly the electron-withdrawing groups (e.g., quinone functional groups) and the electron-donating groups (e.g., phenolic compounds), which significantly contribute to its dual effects on PS-AOPs. In conclusion, NOM has several effects on PS-AOPs, such as activator availability, ROS generation, and OP removal. Given the ubiquitous presence of NOM in the environment, utilizing naturally occurring or externally introduced NOM to enhance its promotional effects on PS-AOPs could lead to more efficient pollutant removal. Further analysis of current research on incorporating NOM into PS-AOPs for OP removal is needed to explore future applications.

Despite the significant achievements made thus far, several challenges remain that must be addressed in future research.

(1) The underlying reaction mechanisms of NOM in PS-AOPs. NOM contains antioxidant functional groups that scavenge free radicals, as well as electron-rich functional groups that enhance the efficiency of PS-AOPs. NOM can play various roles in reactions, including as a reactant, activator, or intermediate. However, the impact of the NOM source, type, and specific functional group composition in complex PS-AOPs is not well understood and requires further investigation. Additionally, the influence of NOM on ROS generation during PS-AOPs in various environments, particularly soil, is still unclear. The effects of traditional probes and quenchers on secondary reaction intermediates and coexisting active substances are not fully understood, highlighting the importance of considering the types and amounts of probes and quenchers used. It may also be beneficial to explore in situ characterization methods for ROS at soil interfaces to obtain more precise information [112,113].
(2) Exploiting suitable methods to characterize the effects of actual NOM. The complicated structure of NOM components and the lack of corresponding characterization methods hinder the study of NOM effects in PS-AOPs. Consequently, model compounds as NOM representatives are usually utilized to simplify the study and obtain clearer and more explicable results [29,47,83]. However, these results may differ from those obtained using actual NOM. Therefore, suitable characterization methods need to be developed to enable a more comprehensive understanding of NOM reactions.
(3) Developing practical applications of PS-AOPs with NOM. Current research is primarily focused on laboratory simulations using batch-reactor systems. However, the oxidation system can be influenced by various factors such as utilization methods, reaction conditions, and environmental composition [14,109]. Therefore, it is essential to comprehensively consider these variable factors based on different situations (e.g., groundwater environments) to maximize the promoting effects of NOM and enhance the efficiency of PS-AOPs. In future studies, it may be beneficial to establish model parameters for the removal of OPs.

## Figures and Tables

**Table 2:** Key groups responsible for the promoting effects of NOM in PS-AOPs.

References	Effects of NOM	Type of NOM	Type of PS-AOPs	Target Pollutant
[83]	Fe(III)-CAT complexes, quinone intermediates, and CAT radicals are involved in ROS generation by generating intermediates and electron transfer.	Catechin (CAT) 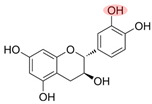	CAT/Fe(III)/PMS	Ofloxacin
[81]	An Fe-TA structure is formed. TA can bind strongly to the carbon substrate through hydrogen bonding, promoting material recombination and improving catalyst performance.	Tannic acid (TA) 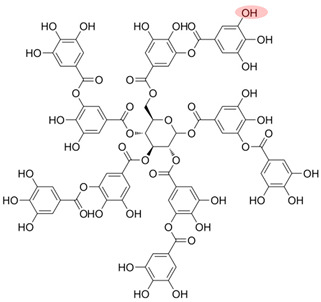	Fe-TCs ^1^/PMS	Bisphenol A
[84]	Fe(II) is reduced to Fe^0^, preventing the aggregation of nanoparticles and promoting dispersion.	Extracted tea polyphenol (TP) 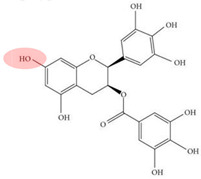	nZVI/PDS	1,2-Dichlorobenzene
[85]	PCA complexes Fe(III) and reduces it to Fe(II). Semiquinones and ortho-quinones generated by PCA conversion promote the conversion of Fe(III)/Fe(II).	Protocatechuic acid (PCA) 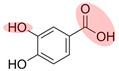	PCA/Fe(II)/PDS	Methyl orange
[79]	Formation of quinone compounds by SA during electron transfer and oxidation promotes the Fe(III)/Fe(II) cycle.	Sinapic acid (SA) 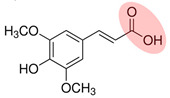	SA/Fe(III)/PMS	Methylparaben
[86]	HA interacts with pollutants and co-adsorbs or accumulates on the catalyst to promote the degradation of carbamazepine. HA activates PMS to some extent.	Humic acid (HA) 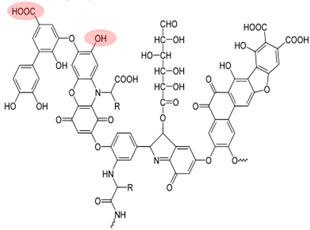	HA/OCN ^2^/PMS	Carbamazepine

Note: Red labels correspond to groups that promote PS-AOPs. ^1^ Fe-TCs: Fe^0^/graphitized carbon composites. ^2^ OCN: Oxygen-doped porous graphite carbon nitride.

## Data Availability

No new data were created or analyzed in this study. Data sharing is not applicable to this article.
